# Prediction of drug-target interactions from multi-molecular network based on LINE network representation method

**DOI:** 10.1186/s12967-020-02490-x

**Published:** 2020-09-07

**Authors:** Bo-Ya Ji, Zhu-Hong You, Han-Jing Jiang, Zhen-Hao Guo, Kai Zheng

**Affiliations:** 1 Xinjiang Technical Institutes of Physics and Chemistry, Chinese Academy of Sciences, Urumqi, 830011 China; 2 University of Chinese Academy of Sciences, Beijing, 100049 China; 3 School of Computer Science and Engineering, Cen-tral South University, Changsha, 410083 China

**Keywords:** Drug-target interactions, Heterogeneous information network, LINE, Random forest

## Abstract

**Background:**

The prediction of potential drug-target interactions (DTIs) not only provides a better comprehension of biological processes but also is critical for identifying new drugs. However, due to the disadvantages of expensive and high time-consuming traditional experiments, only a small section of interactions between drugs and targets in the database were verified experimentally. Therefore, it is meaningful and important to develop new computational methods with good performance for DTIs prediction. At present, many existing computational methods only utilize the single type of interactions between drugs and proteins without paying attention to the associations and influences with other types of molecules.

**Methods:**

In this work, we developed a novel network embedding-based heterogeneous information integration model to predict potential drug-target interactions. Firstly, a heterogeneous multi-molecuar information network is built by combining the known associations among protein, drug, lncRNA, disease, and miRNA. Secondly, the Large-scale Information Network Embedding (LINE) model is used to learn behavior information (associations with other nodes) of drugs and proteins in the network. Hence, the known drug-protein interaction pairs can be represented as a combination of attribute information (e.g. protein sequences information and drug molecular fingerprints) and behavior information of themselves. Thirdly, the Random Forest classifier is used for training and prediction.

**Results:**

In the results, under the five-fold cross validation, our method obtained 85.83% prediction accuracy with 80.47% sensitivity at the AUC of 92.33%. Moreover, in the case studies of three common drugs, the top 10 candidate targets have 8 (Caffeine), 7 (Clozapine) and 6 (Pioglitazone) are respectively verified to be associated with corresponding drugs.

**Conclusions:**

In short, these results indicate that our method can be a powerful tool for predicting potential drug-target interactions and finding unknown targets for certain drugs or unknown drugs for certain targets.

## Introduction

Predicting potential drug-target interactions (DTIs) plays an important part in drug research and discovery. It not only helps researchers better understand biological processes but also reduces the failure rates and costs in the development of new drugs [1, 2]. However, there are still many difficulties in the prediction of drug-target interactions. For example, drugs have many positive and negative effects that are difficult to detect and clarify. In addition, different people respond differently to drugs, even if the gene products are slightly different [3–6]. Moreover, the biological interactions in the human body are extremely complex, making it difficult to trace the effect of drugs. In the past few years, humans have made great efforts in predicting drug-target interactions to overcome these difficulties. With the completion of the Human Genome Project and the development of molecular medicine, more and more unknown drug-target interactions have been discovered. However, due to the high time-consuming, high cost and small research scope of the previous traditional experimental methods, the number of experimentally validated drug-target pairs is still very small. Therefore, this has spurred researchers to develop new computational methods to overcome these limitations to predict potential drug-target interactions [7–9].

At present, a number of public online drug-target interaction databases, such as DrugBank [10], STITCH [11], KEGG [12] and ChEMBL [13], all store the major information about drugs and their interacting targets. These databases greatly facilitate the study of new methods involving drug-target interactions, and many existing calculation models are based on the known drug-target interactions in these databases to predict potential drug-target interactions. More specifically, these methods can be roughly divided into two categories: docking simulation and machine learning. However, the docking simulation method usually requires a three-dimensional (3D) structure of the target (traditional docking) or a larger set of drugs (reverse docking). Because of these limitations of the less known 3D structure of the target or the small size of the existing drug data sets or the high time-consuming, this method is often difficult to conduct. Therefore, machine learning methods are more commonly used in the prediction of drug-target interactions. For example, Wang et al. [14] encoded the protein sequence as a position-specific scoring matrix (PSSM) descriptor to represent biological evolution information of proteins and encoded the drug molecules as a fingerprint feature vector to indicate the presence of a specific functional group or fragment. After that, the Rotation Forest classifier was adapted for the prediction of potential drug-target interactions. Wang et al. [15] used the stacked auto-encoder model in deep learning to fully extract drug molecular structure and protein sequence information. In this way, they generated highly representative features through multiple layers of iteration. Finally, the Rotation Forest classifier was used for the prediction of potential drug-target interactions and achieved good results. Meng et al. [16] developed a novel prediction model for the potential drug-target interactions based on the protein sequence. This method combined position-specific scoring matrix (PSSM), principal component analysis (PCA) with relevance vector machine (RVM) and bi-gram probabilities (BIGP), and had good effectiveness and robustness. Li et al. [17] proposed a computational model for the prediction of drug-target interactions, which used the position-specific scoring matrix (PSSM) of the target protein sequence information, the discriminant vector machine (DVM) classifier, the local binary pattern (LBP) histogram descriptor and the high-identification information of the drug-target interactions. The experimental results show that this method can effectively predict the potential drug-protein interactions. Huang et al. [18] exploited the pseudo substitution matrix representation (Pseudo-SMR) descriptors to represent the protein sequence and used a new fingerprint feature vector to represent the drug signatures. After that, the two vector spaces are connected to represent the drug-protein interaction pairs. The final experimental results indicated that this method has a good performance for the prediction of the potential drug-protein interactions. Wen et al. [19] developed an algorithm framework based on deep learning to predict the potential drug-protein interactions. This approach solves the shortcomings of many traditional methods, which relied heavily on descriptors describing proteins and drugs, and can accurately predict the potential interactions between drugs and targets.

**Fig. 1:**
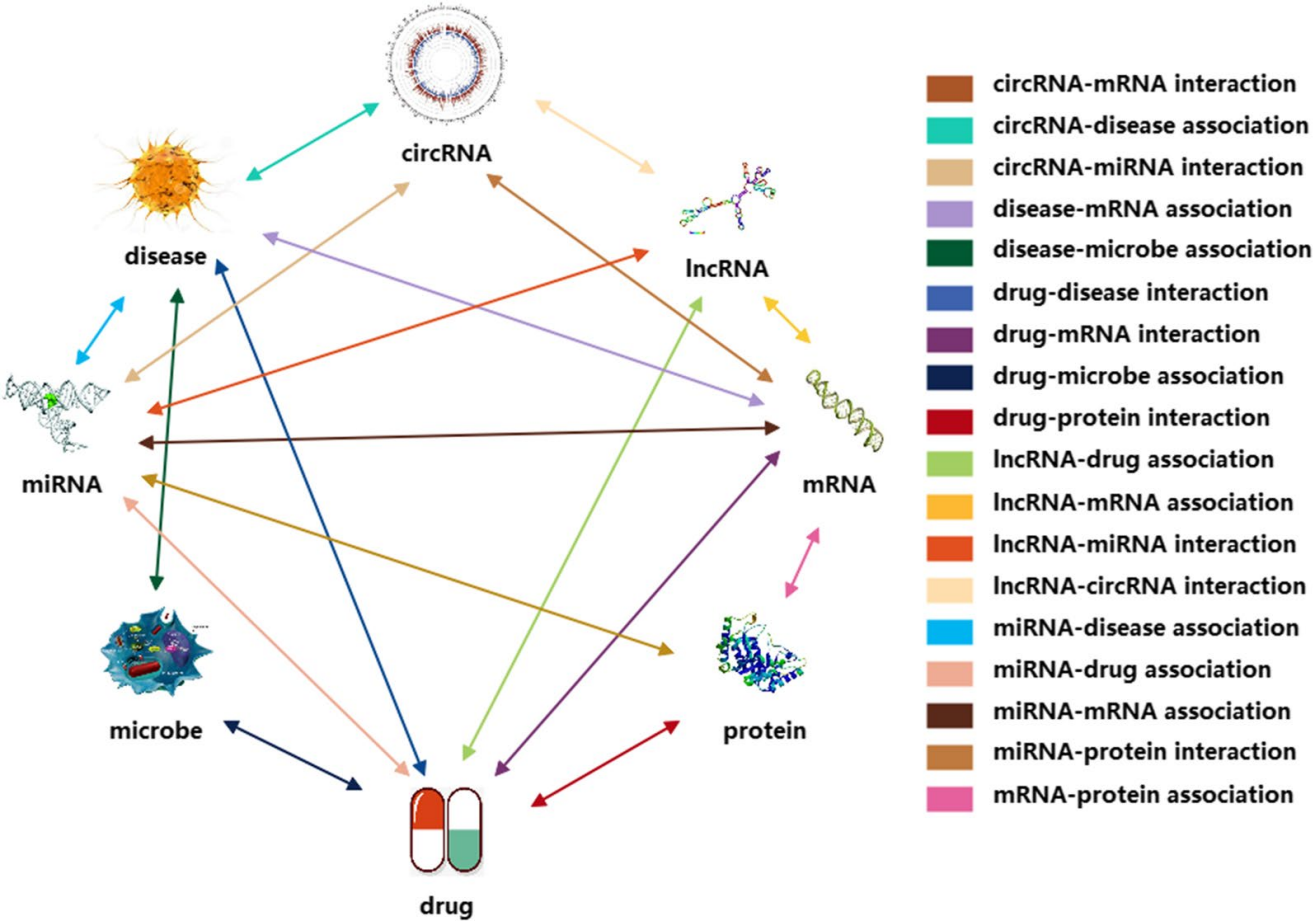
The heterogeneous association information network

**Fig. 2:**
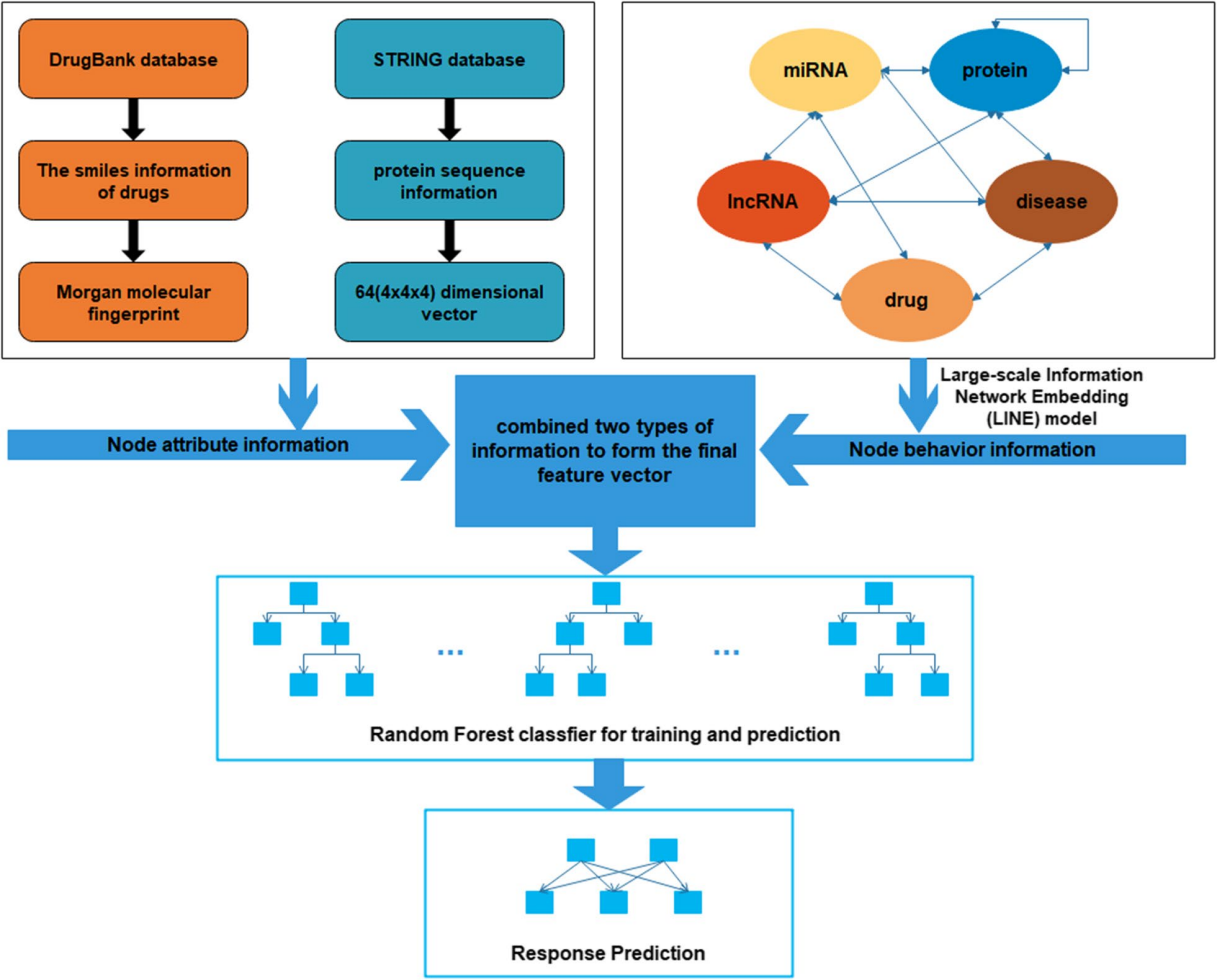
Computation framework of our model

However, many existing computational methods only utilize the single-type of known drug-target association information without paying more attention to the associations between drugs and proteins and other biomolecules. In this work, we propose a novel computational model for predicting potential drug-target interactions. Firstly, we comprehensively analyzed and constructed a heterogeneous multi-molecular information network by combining known associations among disease, protein, drug, lncRNA, and miRNA from multiple databases as shown in Fig. 1. In the network, the nodes and undirected edges among these nodes respectively represent lncRNAs, miRNAs, diseases, drugs and proteins, and interactions among them. In this way, the heterogeneous information network can help people more clearly understand the various life activities of living things [20, 21]. Secondly, the LINE [22] method is conducted to extract the association information between drugs and proteins and other nodes in the network, which we call the behavior information of drugs and proteins. The LINE method can map tightly connected nodes in large networks to similar low-dimensional vector space locations. Thirdly, we integrate the attribute information (sequences of proteins and drugs’ molecular fingerprints) and behavior information (associations with other molecules) to represent known drug-protein interaction pairs. Finally, the Random Forest classifier is applied for the training and prediction of the drug-target interactions. For the training samples in our model, 11107 known drug-protein interaction pairs obtained from DrugBank 3.0 [10] databases are selected as positive sample sets, and the negative sample sets consist of the same number of randomly selected pairs of unrelated drugs and proteins. Figure 2 shows the computation framework of our proposed model. In the results, our method was estimated under the fivefold cross-validation and achieved average the areas under the ROC curve (AUC) and the areas under the PR curve (AUPR) of 0.9233 and 0.9301, respectively. In addition, we also compared the performance of different classifiers and different feature combinations of our method. Besides, in order to further estimate the performance of our model, we also conduct case studies of three major drugs. All these results fully demonstrate that our method has a good performance for drug-target interaction prediction in practical applications.

## Materials and methods

### Combine eight kinds of associations to construct the heterogeneous multi-molecular information network

The heterogeneous association network is composed of known relationships among protein, drug, disease, miRNA, and lncRNA. We download these known associations from multiple databases and unify identifiers, remove redundant items, simplify and delete unrelated items. The final detailed data is shown in Table 1. In addition, we further counted the number of each node in the network. The final statistical results are shown in Table 2.

**Table 1:** The association information in the network

Association	Database	Amount
miRNA-lncRNA	lncRNASNP2 [23]	8374
miRNA-disease	HMDD v3.0 [24]	16,427
miRNA-protein	miRTarBase:update 2018 [25]	4944
LncRNA-disease	LncRNADisease [26],	
	lncRNASNP2 [23]	1264
Drug-disease	CTD: update 2019 [27]	18,416
LncRNA-protein	LncRNA2Target v2.0 [28]	690
Protein–protein	STRING: in 2017 [29]	19,237
Protein-disease	DisGeNET [30]	25,087
Total	N/A	94,439

**Table 2:** The node information in the network

Node	Amount
Drug	134
MiRNA	1023
Disease	2062
Protein	613
LncRNA	769
Total	4601

### Drug molecular fingerprint

The Simplified Molecular Input Line Entry Specification (SMILES) of drugs mainly utilizes letters and symbols to indicate the structure of the compound for computer input. It is very different from traditional chemical formulas and has special writing rules. We download the drug’s smiles from the DrugBank 3.0 [10] database and then convert the drug’s smile to the relevant Morgan Molecular Fingerprint through using the RDKit python package.

### Protein sequence information

The protein sequence information is derived from the STRING [29] database and used to represent the attribute information of the protein. After that, we choose the method in the article by Shen et al. [31] to encode them. In this paper, according to the polarity of the side chain, 20 amino acids are divided into four categories including (Arg, Lys, and His); (Gly, Cys, Ser, Gln, Thr, Asn, and Tyr); (Ala, Ile, Trp, Val, Leu, Phe, Pro and Met); (Glu and Asp). In this way, each protein sequence can be represented as a 64-dimensional vector, and each dimension denotes the occurrence frequency of a 3-mer (e.g. UCC, AGU).

### Large-scale information network embedding (LINE)

As a novel network embedding method, LINE [32] mainly solves the problem of embedding large information networks into low-dimensional vector spaces. It can map closely connected nodes in a large network to similar low-dimensional vector space positions and is fully used for visualization, node classification, and link prediction. The LINE method is suitable for any type of information network and optimizes a well-designed objective function to retain both local and global network structure information. It not only considers the first-order proximity of nodes, that is, two points are directly connected with an edge of higher power value, they are considered to be more similar, but also considers the second-order proximity of nodes, that is, two points may not be directly connected but is considered similar if they have more public first-order proximity friends. Based on these two perspectives, the LINE model can be divided into the following two categories:

Model 1: LINE with First-order Proximity

It should be noted that this model is only applicable to undirected graphs. For an undirected edge (*i, j*), the joint probabilities of the two vertex $v_{i}$ and $v_{j}$ defining this edge is as follows:

1 $$p_{1} \;\left( {v_{i} ,\,v_{j} } \right)\; = \;\frac{1}{{1 + { \exp }\left( { - \vec{u}_{i}^{T} \vec{u}_{j} } \right)}}$$

where $\vec{u}_{i}$ and $\vec{u}_{j}$ are the low-dimensional vector representation of vertex $v_{i}$ and $v_{j}$. It is equivalent to describe the intimacy between vertices from the perspective of embedding. Formula (1) defines the distribution *p*(*,*) on the space V × V, and its empirical probability can be defined as:

2 $$\hat{p}_{1} \;\left( {i,\;j} \right)\; = \;\frac{{{\text{w}}_{\text{ij}} }}{\text{W}}$$

where $w_{ij}$ represents the weight of the edge between vertex $v_{i}$ and $v_{j}$, and W represents the sum of all weights of edges in the network. Our optimization goal is to make the difference between $p_{1}$ and $\hat{p}_{1}$ as small as possible, so the objective function can be defined as follows:

3 $$O_{1} \; = \;d\left( {p_{1} \left( {*,*} \right),\; \hat{p}_{1} \left( {*,*} \right)} \right)$$

where *d()* function is used to measure the difference between the two distributions. Generally, the Kullback–Leibler (KL) divergence can be selected to replace the *d*(*,*). In this way, the KL divergence is brought into the above formula, and the constants can be omitted (e.g. W), the final optimized form can be obtained:

4 $$O_{1} \; = - \mathop \sum \limits_{{\left( {i,j} \right) \in E}} w_{ij} logp_{1} \left( {v_{i} ,v_{j} } \right)$$

Therefore, we can represent each vertex in the *d*-dimensional space by finding the $\left\{ {{\vec{\text{u}}}_{\text{i}} } \right\}_{{{\text{i}} = 1 \ldots \left| {\text{V}} \right|}}$ which minimizes the objective in Eq. (4).

Model 2: LINE with Second-order Proximity

This model considers the effects of second-order relationships between nodes and is suitable for both directed and undirected graphs. For a directed edge (*i, j*) (from *i* to *j*), the probability that vertex $v_{j}$ is a neighbor of $v_{i}$ can be represented as follows:

5 $$p_{2} \;\left( {v_{j} |v_{i} } \right)\; = \;\frac{{{ \exp }\left( {\vec{u}_{j}^{'T} \cdot \vec{u}_{i} } \right)}}{{\mathop \sum \nolimits_{k = 1}^{\left| v \right|} { \exp }\left( {\vec{u}_{k}^{'T} \cdot \vec{u}_{i} } \right)}}$$

where $\left| V \right|$ represents the number of vertices. Next, in order to make the conditional distribution of context $p_{2} \left( { \cdot |v_{i} } \right)$ specified by the low-dimensional representation be closed to the empirical distribution $\hat{p}_{2} \left( { \cdot |v_{i} } \right)$, which is defined as follows:

6 $$\hat{p}_{2} \;\left( {v_{j} |v_{i} } \right)\;{ = }\;\frac{{w_{ij} }}{{d_{i} }}$$

where $d_{i}$ represents the out-degree of vertex *i* and $w_{ij}$ represents the weight of the edge, it is necessary to minimize the following formula:

7 $$O_{2} \; = \;\sum\limits_{i \in V} {\alpha_{i} d\left( {\hat{p}_{2} \left( {*,*} \right),p_{2} \left( { *, *} \right)} \right)}$$

where $\alpha_{i}$ represents the prestige of vertex *i* and can be measured by the degree or estimated through an algorithm such as PageRank [33]. In this article, for convenience, we set $\alpha_{i}$ as the degree of vertex *i* and replace d(*,*) with KL-divergence. The Eq. (7) can be finally optimized as follows:

8 $$O_{2} \; = \;\sum\limits_{{\left( {i,\;j} \right) \in E}} {w_{ij} logp_{2} } \left( {v_{j} \left| {v_{i} } \right.} \right)$$

Therefore, we can represent each vertex $v_{i}$ with a *d*-dimensional vector $\vec{u}_{i}$ via learning $\left\{ {{\vec{\text{u}}}_{\text{i}} } \right\}_{i = 1 \ldots \left| V \right|}$ and $\left\{ {\vec{u}_{i}^{'} } \right\}_{i = 1 \ldots \left| V \right|}$ which minimizes this objective.

### The Receiver Operating Characteristic (ROC) and Precision-Recall (PR) curve

The Receiver Operating Characteristic (ROC) curve is a very important and common statistical analysis method. It sorts and predicts samples according to the prediction results of the classifier. In addition, it calculates the values of two important quantities each time: True Positive Rate (TPR) and False Positive Rate (FPR), which are respectively plotted on the horizontal and vertical coordinates. The AUC value is defined as the areas under the ROC curve and can be used as a numerical value to intuitively evaluate the quality of the classifier. Generally, the larger the AUC value, the more accurate the prediction result and the better the classification effect of the model. The Precision-Recall (PR) curve is also a method to test the capability of a classifier. Compared with the ROC curve, the PR curve can better reflect the performance of the classification when the proportion of positive and negative samples is large.

### Node representation

Drugs and proteins are respectively represented by attribute information and behavior information (association information with other molecules) in the network we constructed. Their attribute information is respectively sequences of proteins and molecular fingerprints of drugs. Besides, in this article, we choose a network embedding model LINE to get the behavior information of them. In this way, the final 128-dimensional feature vector contains 64-dimensional attribute information (protein sequences information and drug molecular fingerprints) and 64-dimensional behavior information (associations with other molecules) of drugs and targets. These two types of information are functionally similar and collaboratively provide information for the classifier to predict the potential associations between drugs and targets.

## Result and discussion

### Evaluation of our model under fivefold cross validation

Cross-validation is a statistical analysis method for verifying the performance of a classifier to obtain a reliable and stable model. In this work, fivefold cross-validation is conducted to estimate the performance of our model. 11107 known drug-target interaction pairs obtained from DrugBank 3.0 [10] database are used as training samples. In this way, we take 4/5 samples (training set) to build the model and leave 1/5 sample (test set) to predict the newly built model. We repeat this experiment 5 times so that the model can effectively avoid over- or under-learning, and the results obtained are more persuasive. In this article, we choose the following six common parameters as the evaluation indicators of our model: Accuracy (Acc.), Specificity (Spec.), Sensitivity (Sen.), Precision (Prec.), Matthews Correlation Coefficient (MCC), Areas under the ROC Curve (AUC). The detailed results of our method are shown in Table 3, and the last row of Table 3 shows the average value and their standard deviation of the results across 5 runs of the classifier.

**Table 3:** Evaluation of our model under five-fold cross-validation

Fold	ACC. (%)	Spec. (%)	Prec. (%)	MCC (%)	Sen. (%)	AUC (%)
0	86.45	91.49	90.54	73.28	81.41	92.90
1	85.87	90.86	89.85	72.10	80.87	92.31
2	85.08	90.82	89.63	70.63	79.34	92.05
3	85.13	91.27	90.05	70.79	78.98	91.71
4	86.64	91.53	90.61	73.63	81.75	92.66
Average	85.83±0.72	91.19±0.34	90.14±0.43	72.09±1.38	80.47±1.24	92.33±0.47

**Fig. 3:**
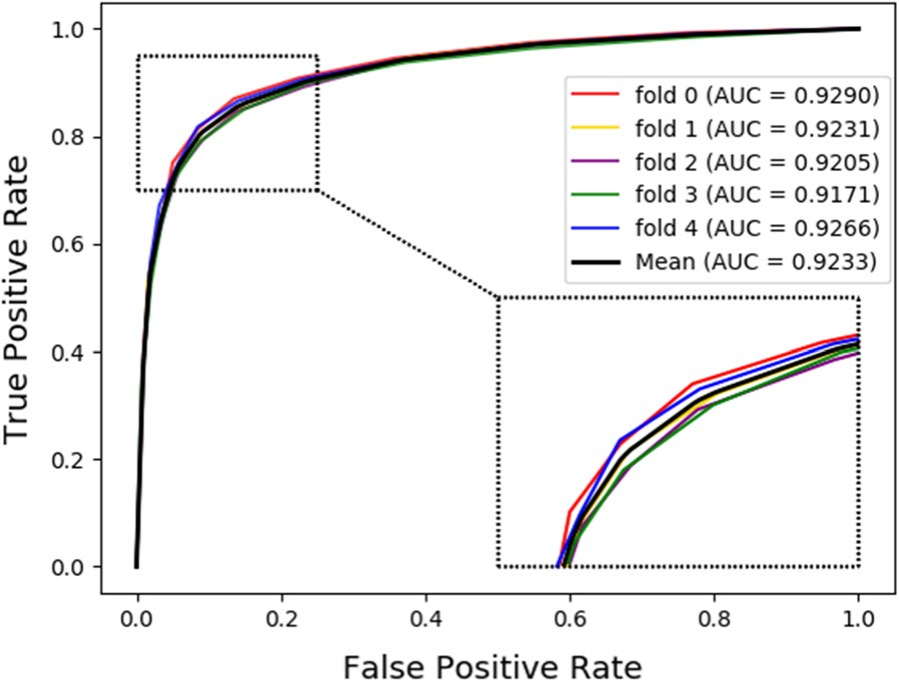
The ROC curves of our model under fivefold cross-validation

**Fig. 4:**
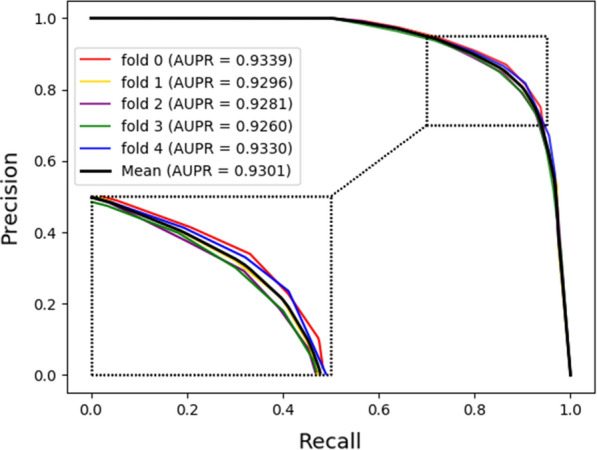
The PR curves of our model under five-fold cross-validation

Figures 3, 4 respectively show the ROC curves and AUC values, PR curves and AUPR values of our model under five-fold cross validation. It can be seen from the figure that the mean AUC and AUPR of our model are 0.9233 and 0.9301, respectively. The results fully demonstrate that our proposed model has a good performance for potential drug-target interactions prediction. Besides, the variance of a model can describe the generalization ability of it. Generally, the larger the variance, the easier the model is disturbed. On the contrary, the smaller the variance, the more stable the model. In this work, the variance of the AUC for 5 runs of our model is 0.002%. The small variance can also prove that our method is stable for the prediction of potential drug-target interactions.

### Comparison of different feature combinations

**Fig. 5:**
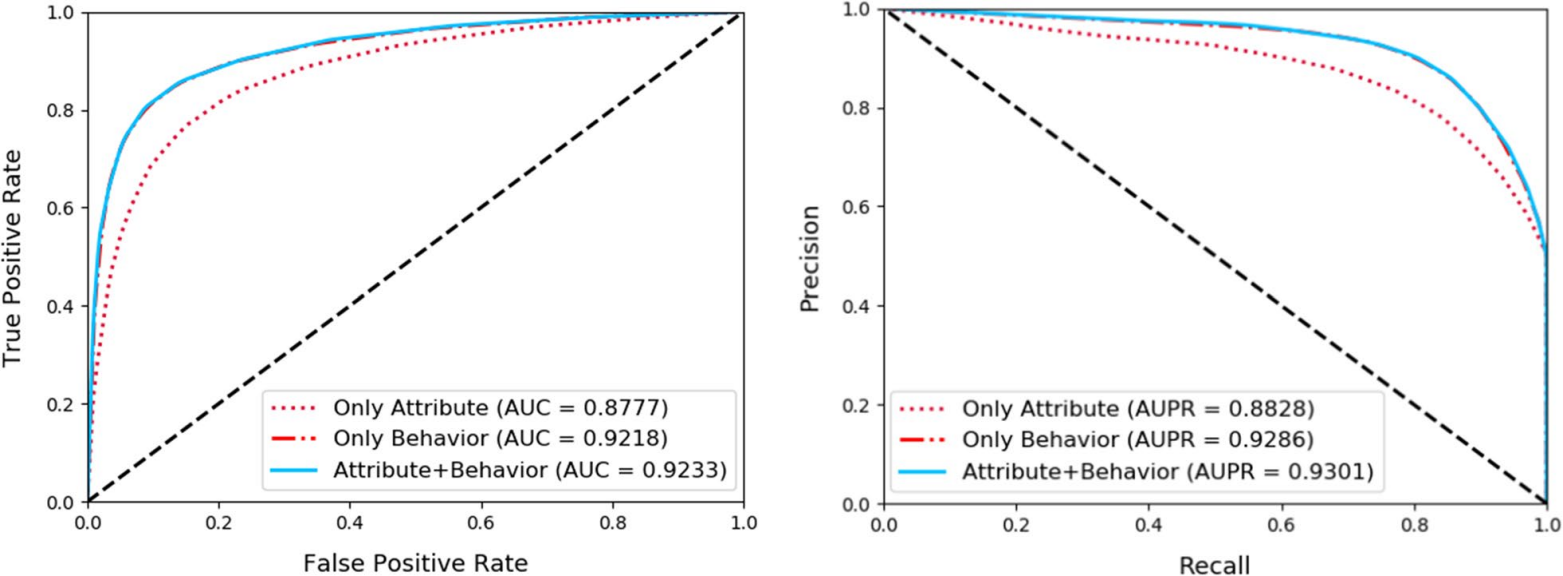
Comparison of different feature combinations under fivefold cross validation

As we mentioned before, our approach utilizes a combination of attribute and behavior information to represent known drug-protein interaction pairs. Hence, to test the performance of different feature combinations on the results, we further conducted experiments with three different feature combinations. More specifically, we use only attribute information, only behavior information, and the combination of attribute and behavior information to respectively represent the drug and protein nodes. After that, the fivefold cross-validation experiment was conducted respectively. The experimental environment and parameters of the three modes are consistent. Table 4 and Fig. 5 show the detailed results of three models, and the classification results are better when we utilize both the attribute and behavior information.

**Table 4:** Comparison of different feature combinations

Feature	Acc. (%)	Spec. (%)	Prec. (%)	MCC (%)	Sen. (%)	AUC (%)
Attribute	80.73 ± 0.79	84.36 ± 1.05	83.14 ± 1.04	61.63 ± 1.61	77.11 ± 0.60	87.77 ± 0.83
Behavior	85.75 ± 0.59	91.12 ± 0.90	90.06 ± 0.92	71.92 ± 1.21	80.37 ± 0.68	92.18 ± 0.51
Both	85.83 ± 0.72	91.19 ± 0.34	90.14 ± 0.43	72.09 ± 1.38	80.47 ± 1.24	92.33 ± 0.47

### Comparison of different machine learning classifiers

**Fig. 6:**
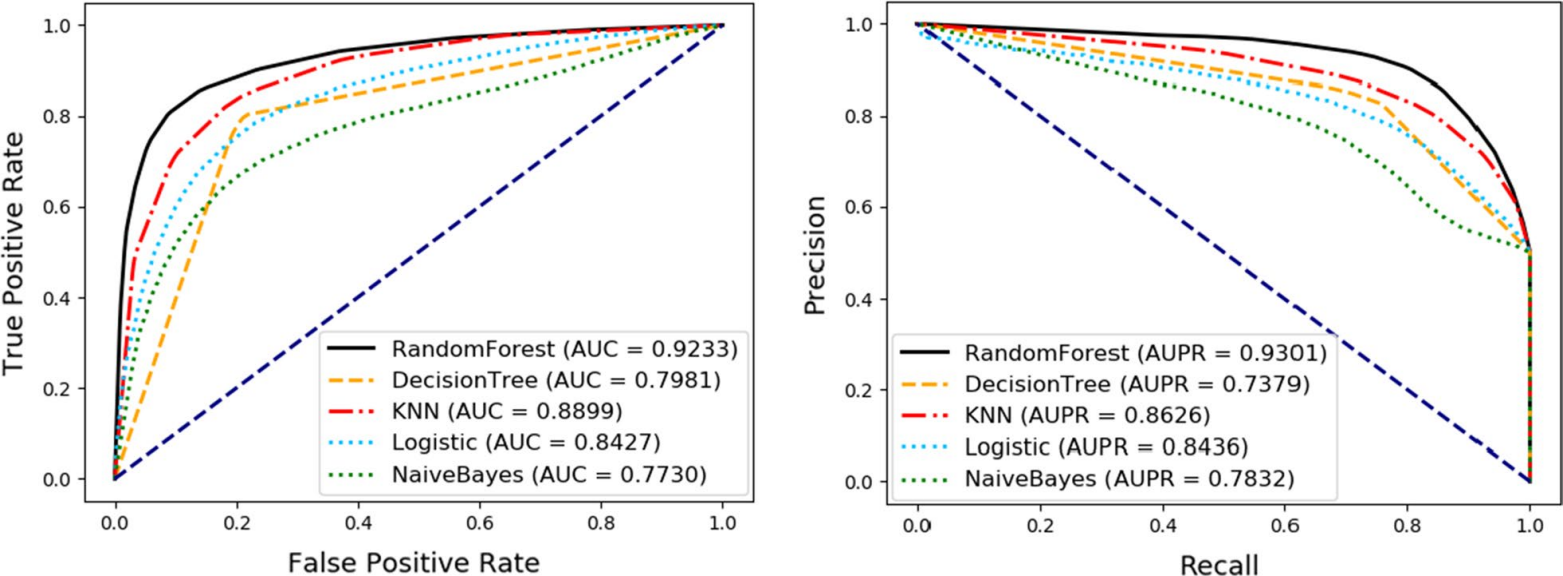
Comparison of different machine learning classifiers under fivefold cross-validation

To estimate the impact of different classifiers on the final results, we further respectively use Logistic, KNN, Naive Bayes, Decision Tree, and Random Forest classifier to perform fivefold cross-validation on our proposed model. In particular, all the variables in the experiment are the same for the five classifiers, and all the classifiers use default parameters to make the comparative results more fair and reliable. The detailed results can be founded in Table 5 and Fig. 6. As can be seen from the results, the Random Forest classifier is not as good as KNN in sensitivity, but it has better performance for AUC and accuracy, which can better reflect the performance of our model. In conclusion, the Random Forest has a better performance than other classifiers and is more suitable for our method.

**Table 5:** Comparison of different machine learning classifiers

Classifier	ACC. (%)	Spec. (%)	Prec. (%)	MCC (%)	Sen. (%)	AUC (%)
Logistic	77.63 ± 1.03	81.19 ± 0.75	79.74 ± 0.91	55.40 ± 2.04	74.06 ± 1.41	84.27 ± 1.30
KNN	82.04 ± 1.19	79.83 ± 2.26	80.72 ± 1.74	64.15 ± 2.32	84.24 ± 0.78	88.99 ± 0.81
Naive Bayes	72.57 ± 1.16	73.74 ± 1.09	73.11 ± 1.04	45.15 ± 2.31	71.39 ± 1.91	77.30 ± 1.57
DecisionTree	79.81 ± 0.66	79.73 ± 1.29	79.78 ± 1.01	59.63 ± 1.32	79.89 ± 0.60	79.81 ± 0.66
RandomForest	85.83 ± 0.72	91.19 ± 0.34	90.14 ± 0.43	72.09 ± 1.38	80.47 ± 1.24	92.33 ± 0.47

### Case studies

To further estimate the performance of our model in practical applications, we select three common drugs (Caffeine, Clozapine, and Pioglitazone) for case studies. These three drugs are all closely related to human health and are often chosen by many computational methods for case studies.

The chemical composition of Caffeine is 1,3,7-trimethylamine, which can be founded in tea, coffee, cocoa, guarana and kola [34]. Recently, many researches have been reported that caffeine may have an anti-cancer effect [35–37] and orally applied caffeine can protect the skin from skin cancer caused by ultraviolet (UV) rays [38, 39]. Besides, transdermally applied caffeine can be used to treat skin cancer locally and systemically.

Clozapine is a second-generation psychiatric drug. In addition, it has been proved that clozapine is effective for psychotic positive and negative symptoms. Contrary to concerns that typical antipsychotics may aggravate drug abuse, recent reports indicate that clozapine has a reduced effect on nicotine, alcohol or other drug abuse in patients with schizophrenia [40, 41]. Clozapine can also alleviate the emotional symptoms associated with schizophrenia (depression, guilt, anxiety), as well as the excitement and illusion of treatment for mania or other psychotic disorders.

Pioglitazone is a hypoglycemic drug that can be used alone or in combination with other hypoglycemic agents for the treatment of type 2 diabetes. The main function of this medicine is to reduce the insulin resistance in the body and enhance the sensitivity of the cells to insulin so that the body can make full use of the existing insulin to achieve the purpose of lowering blood sugar. At the same time, pioglitazone can improve the blood fat and pressure of the patient and reduce the blood vessels of the heart [42]. The drug has been well-tolerated by adult patients of all ages in clinical studies [43].

Therefore, the identification of these three drugs’ targets is of great importance. More specifically, we utilize the known drug-protein interactions in the DrugBank 3.0 database of Knox et al. [10] as the training data set in the case studies. One important fact that must be noted is that the known associations with the corresponding drug have been removed from the training data set to illustrate the applicability of our method to new drugs (drugs with no known related proteins). For the test data set, it contains proteins and corresponding drug interaction pairs in the heterogeneous association information network. After the prediction is complete, we rank all the proteins based on the predicted association scores and select the top 10 predicted targets to validate them using two databases on the relationship between drug and target, SuperTarget [44] and DrugBank 5.0 [45].

Table 6 shows the prediction result of the top 10 targets associated with caffeine, and 8 of which were successfully confirmed by the database. For example, the interaction between cytochrome P450 1A2 (CYP1A2) and caffeine has been confirmed by previous experiments [46]. The experiment proves that there is an interaction between caffeine and CYP1A2 by studying the expression of CYP1A2 in mouse striatum.

**Table 6:** Prediction of the top 10 targets associated with Caffeine

UniProt ID	Target	Evidence
9606.ensp00000342007	Cytochrome P450 1A2	SuperTarget
9606.ensp00000360372	Cytochrome P450 2C19	Unconfirmed
9606.ensp00000337915	Cytochrome P450 3A4	SuperTarget
9606.ensp00000478255	ATP-dependent translocase ABCB1	DrugBank
9606.ensp00000360317	Cytochrome P450 2C8	SuperTarget
9606.ensp00000260682	Cytochrome P450 2C9	SuperTarget
9606.ensp00000324648	Cytochrome P450 2B6	Unconfirmed
9606.ensp00000440689	Cytochrome P450 2E1	SuperTarget
9606.ensp00000353820	Cytochrome P450 2D6	SuperTarget
9606.ensp00000222982	Cytochrome P450 3A5	SuperTarget

Table 7 shows the prediction result of our method of the top 10 targets associated with clozapine, 7 of which were successfully confirmed by the database. For example, the interaction between cytochrome P450 1A2 and clozapine has been confirmed by previous experiments [47].

**Table 7:** Prediction of the top 10 targets associated with Clozapine

UniProt ID	Target	Evidence
9606.ensp00000478255	ATP-dependent translocase ABCB1	DrugBank
9606.ensp00000342007	Cytochrome P450 1A2	SuperTarget
9606.ensp00000360372	Cytochrome P450 2C19	SuperTarget
9606.ensp00000260682	Cytochrome P450 2C9	SuperTarget
9606.ensp00000337915	Cytochrome P450 3A4	SuperTarget
9606.ensp00000324648	Cytochrome P450 2B6	Unconfirmed
9606.ensp00000353820	Cytochrome P450 2D6	SuperTarget
9606.ensp00000222982	Cytochrome P450 3A5	SuperTarget
9606.ensp00000295897	Serum albumin	Unconfirmed
9606.ensp00000480571	Cytochrome P450 3A7	Unconfirmed

Table 8 shows the prediction result of the top 10 targets associated with pioglitazone using our method, 6 of which were successfully confirmed by the database. For example, the interaction between cytochrome P450 3A4 and pioglitazone has been confirmed by previous experiments [48]. This study evaluated the effect of pioglitazone on the activity of cytochrome P450 3A4 (CYP3A4), demonstrating that pioglitazone has a concentration-dependent inhibitory effect on CYP3A4 enzyme activity.

**Table 8:** Prediction of the top 10 targets associated with Pioglitazone

UniProt ID	Target	Evidence
9606.ensp00000337915	Cytochrome P450 3A4	SuperTarget
9606.ensp00000478255	ATP-dependent translocase ABCB1	Unconfirmed
9606.ensp00000353820	Cytochrome P450 2D6	SuperTarget
9606.ensp00000367102	Solute carrier family 22 member 6	Unconfirmed
9606.ensp00000222982	Cytochrome P450 3A5	Unconfirmed
9606.ensp00000260682	Cytochrome P450 2C9	SuperTarget
9606.ensp00000360372	Cytochrome P450 2C19	DrugBank
9606.ensp00000369050	Cytochrome P450 1A1	Unconfirmed
9606.ensp00000360317	Cytochrome P450 2C8	SuperTarget
9606.ensp00000256958	Solute carrier organic anion transporter family member 1B1	DrugBank

## Conclusion

The prediction of drug-target (protein) interactions is an important part of understanding the biological process and detecting new drugs. In this work, we put forward a novel network embedding-based heterogeneous information integration model for drug-target interaction prediction. More specifically, we utilize the network representation method LINE to obtain the behavior information (associations with other nodes) of drug and protein node in the network and then combine it with the intrinsic attribute information of them to represent the known drug-protein interaction pairs. Finally, the Random Forest classifier is selected to train and predict the transformed feature vectors. As a result, our proposed method has good performance for the potential drug-target interactions prediction under the five-fold cross-validation, and the prediction results are better than the model of using only behavior information or attribute information. Besides, to further estimate the performance of our model, we also conduct case studies of three common drugs (Caffeine, Clozapine, and Pioglitazone). The results of case studies further indicate that our model performs well in predicting the potential drug-target interactions and targets associated with a given drug. Generally speaking, our proposed model can be an efficient tool for the prediction of potential drug-target interactions in the future.

## Data Availability

The datasets analyzed during the current study are available from the corresponding author on reasonable request.
